# Advantages of Multidetector-Row Computed Tomography for Detecting Transverse Mesocolic Internal Hernia

**DOI:** 10.2174/0115734056359062250414074213

**Published:** 2025-04-28

**Authors:** Le Duc Nam, Thai Khac Trong, Nguyen Van Thach, Le Duy Dung, Lam Sao Mai, Tong Thi Thu Hang

**Affiliations:** 1 Center of Diagnostic Imaging, Military Central Hospital 108, Hanoi, Vietnam; 2 CATS Academy Boston, Boston, USA

**Keywords:** Abdominal internal hernia, Transmesenteric hernia, Closed-loop intestinal obstruction, Multidetector-row computed tomography, Internal hernia, Laparoscopic surgery

## Abstract

**Introduction:**

A transverse mesocolic internal hernia is a phenomenon in which a small intestinal loop protrudes through the natural orifice in the transverse colon mesentery. This type of internal hernia in adults, although rare, is one of the causes of closed-loop intestinal obstruction, which requires prompt diagnosis and treatment.

**Case Presentation:**

We report two cases of transverse mesocolic internal hernia that were examined and subsequently treated at Hospital 108, Hanoi, Vietnam. Both patients (53 and 66 years old) had atypical clinical symptoms, mainly dull epigastric pain. Upon admission, they were initially examined clinically, followed by blood testing and chest and abdominal X-ray radiography. Diagnostic imaging was mainly based on subsequent Multidetector-Row Computed Tomography (MDCT). Laparoscopic/surgical release of the hernia and closure of the natural orifice in the transverse colon mesentery were performed. The clinical symptoms and laboratory and radiographic findings did not suggest a causal diagnosis. However, MDCT provided several images suggestive of an internal hernia, including a closed intestinal loop passing through the transverse colon mesentery and located posteriorly in the left abdominal cavity near the Treitz angle, displacement of the mesenteric vascular bundle, and colon displacement. These displacements were the causes of intestinal inflammation/obstruction. Additionally, laparoscopic/surgical results confirmed the MDCT diagnosis.

**Conclusion:**

Thin-slice thickness, high spatial resolution, multiplanar reconstruction MDCT was effective for diagnosing transverse mesocolic internal hernia. In our two cases, MDCT helped determine the cause and assess the state of intestinal ischemia.

## 1 INTRODUCTION

Internal abdominal hernia is the abnormal movement of abdominal organs (mainly small intestinal loops) through natural orifices or defects in the peritoneum or mesentery, congenital or acquired [1, 2]. Internal hernia through natural orifices in the Transverse Colon Mesentery (TCM) occurs in 0.5% of autopsy cases, accounting for approximately 8% of internal hernia cases [3-5]. The clinical picture of internal hernia, in general, and transverse mesocolic internal hernia, in particular, is often unclear, which often leads to late or misdiagnosis that can result in serious sequelae. Since its inception, Multidetector-Row Computed Tomography (MDCT) has become an effective imaging technique for diagnosing internal hernias. The thin-slice thickness, high spatial resolution, and multiplanar reconstruction capabilities of MDCT provide detailed information about structures within the abdominal cavity and their condition. This contributes significantly to accurate diagnosis, predicting severity, and proposing appropriate treatment for the patient [6].

We report two cases of transverse mesocolic internal hernia, localized in the left abdominal cavity near the angle of Treitz, with complications of intestinal inflammation/ obstruction. We also review the relevant literature to provide more examples of the role of MDCT in diagnosing this type of internal hernia.

## 2 CASE SERIES

### 2.1 Case 1

A 53-year-old male patient had a history of good health and no abdominal surgery. Approximately 2 days before admission, the patient experienced dull epigastric pain, accompanied by a fever of 39°C, with normal urination and defecation and no vomiting.

#### 2.1.1 Examination Upon Admission

 The patient was conscious and had a fever of 38°C. His hemodynamics were stable, and his abdomen was soft with no distension, though he was experiencing epigastric pain without an abdominal wall reaction. There was no sign of snake crawling.

#### 2.1.2 Hematological Tests

White blood cell count was elevated at 21.1 G/L, with neutrophils at 78.5%, while other tests were normal. Chest and abdominal X-ray radiography showed no abnormalities.

**Fig. (1):**
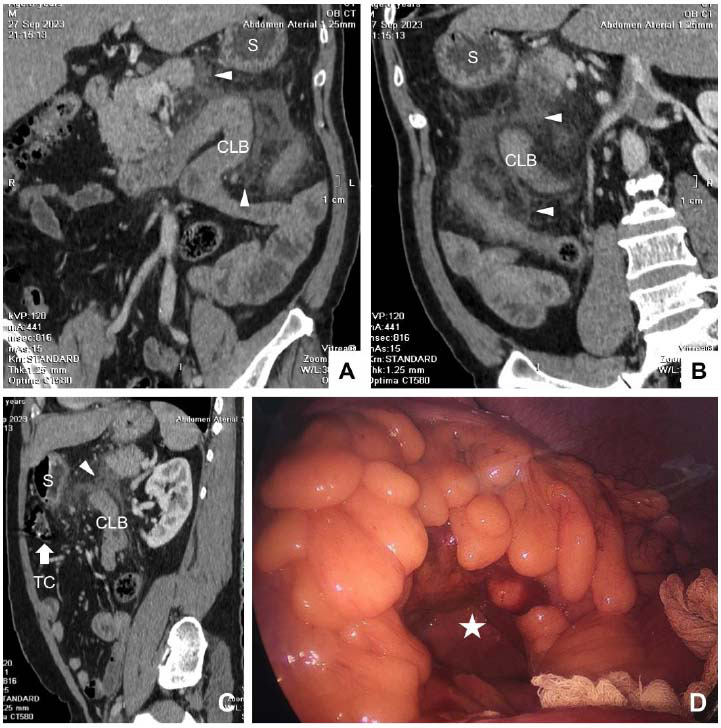
Multidetector-row computed tomography (MDCT) **(A-C)** and laparoscopic **(D)** images of a 53-year-old male patient experiencing dull epigastric pain. The MDCT images on the coronal **(A)** and oblique **(B, C)** planes showed a U-shaped loop of the small intestine, passing through the transverse colon (TC) mesentery near the angle of Treitz, that formed a hernia sac located behind the TC (white arrow), measuring 5.5 × 9.5 cm, with a thickened intestinal wall (9.3 mm), infiltration of adjacent fat tissue (arrowheads) in the hernia sac and at the root of the loop mesentery, suggesting localized enteritis, with no free fluid or air in the abdominal cavity. **D:** Laparoscopy of the TC mesentery showing an approximately 4 cm in diameter natural orifice (a white star) and 2 cm deep, with the first loop of the jejunum passed through it and signs of adhesion. CLB = Closed loop of bowel, S = Stomach, TC = Transverse colon.

MDCT images showed that there was a loop of the small intestine passing through the TCM near the angle of Treitz, with thickening of the intestinal loop wall (9.3 mm), infiltration of adjacent fatty tissue in the hernia sac and at the mesenteric root of the intestinal loop, suggesting localized enteritis, and no free fluid or air was seen in the abdominal cavity. We performed a laparoscopy to identify the cause of his epigastric pain. The endoscopic images were consistent with the MDCT images. Specifically, the endoscopy showed a natural orifice of 4 cm in diameter and 2 cm in depth in the TCM. The first jejunal loop passed through it and showed inflammation, adhesion, and infiltration of adjacent fatty tissue (Fig. **1**). The surgeon removed the jejunal loop from the above-mentioned orifice and then closed it to prevent hernia recurrence. No signs of intestinal ischemia were observed. There were no complications during and after surgery, and the patient was discharged on post-endoscopy Day 5.

### 2.2 Case 2

A 66-year-old male patient had a history of good health and no abdominal surgery. Approximately 10 days before admission, the patient felt dull epigastric abdominal pain accompanied by a burning sensation. Outpatient examination and gastroscopy detected antral gastritis. Medication was prescribed, but little improvement was observed. Approximately 3 days before admission, the patient experienced increased pain and nausea but was still able to pass gas. His vital signs were normal as follows: Temperature 37ºC; heart rate, 75 beats/minute; blood pressure, 140/70 mmHg; and respiratory rate, 20 breaths/minute. The general examination showed no specific signs, so he was diagnosed with a gastric ulcer. However, treatment for an ulcer did not improve his abdominal pain. At admission, an abdominal examination revealed distension, left-flank pain, crystal-clear percussion, and high-pitched bowel sounds, suggesting high intestinal obstruction syndrome. Blood testing showed a slight increase in white blood cells (13.13 G/L), and neutrophils accounted for 75.3%. Other test results were normal. Chest X-ray radiography showed no abnormalities, and abdominal X-ray showed a dilated stomach and small intestine, suggesting a high intestinal obstruction. Initially, the patient was conservatively treated with intravenous fluids and gastric-tube placement; subsequently, 1500 ml of dirty digestive fluid was seen. However, his clinical symptoms did not improve, so an MDCT scan was scheduled.

**Fig. (2):**
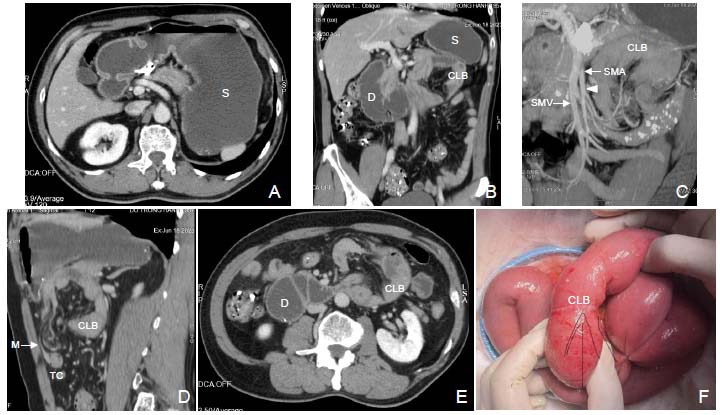
Multidetector-row computed tomography (MDCT) (**A-E**) and intraoperative (**F**) images of a 66-year-old male patient with dull epigastric pain. Axial (**A, E**) and coronal (**B**) CT images showing a dilated stomach (S) and duodenum (D) filled with fluid, suggesting a high intestinal obstruction. Maximum intensity projection (MIP) (**C**) and sagittal (**D**) CT images showing that the superior mesenteric artery (SMA) and vein (SMV) vascular bundle is pushed to the right (white arrowhead), and the transverse colon is pushed forward by the closed loop of the bowel (CLB). Sagittal (**D**) and axial (**E**) images show an inverted C-shaped first loop of the small intestine, herniated through the transverse colon mesentery, and located in the left-abdominal cavity superior-posterior to the transverse colon (TC) near the angle of Treitz. No signs of intestinal ischemia and no free fluid or air are apparent in the abdominal cavity. Intraoperative image (**F**) showing that the first loop of the small intestine was strangulated at the natural orifice in the transverse colon mesentery; however, the surface of the loop was still pink, with no signs of ischemia or necrosis. CLB = Closed loop of bowel, D = Duodenum, M = Transverse mesentery, S = Stomach, SMA = Superior mesenteric artery, SMV = Superior mesenteric vein, TC = Transverse colon.

The MDCT scan showed that in the left abdomen near the angle of Treitz, there was an inverted C-shaped loop of the small intestine passing through the TCM, and the second part of the duodenum was dilated, suggesting that there was blockage of the high digestive tract. The mesenteric vascular bundle (superior mesenteric artery and vein) was pushed to the right, and the TC was pushed forward by the closed loop of the bowel. There was no sign of intestinal ischemia and no free fluid or air in the abdomen (Fig. **2**). The diagnosis of MDCT was high intestinal obstruction due to an internal hernia through the TCM. The patient underwent laparoscopy to determine the cause, which showed that a small intestinal loop passed through a natural orifice in the TCM where it was strangulated; however, the intestinal wall was still pink, and there was no sign of anemia or necrosis. An attempted laparoscopic release of the intestinal loop was unsuccessful, so a decision was made to perform open surgery. During the surgery, the jejunal loop was pulled down to free it from the hernia, the hernia orifice was sutured, and the first two intestinal loops near the duodenum-jejunal angle were sutured to shorten the mesentery of the small intestine. The surgery was accident- and complications-free, and there were no postoperative complications. The patient was discharged on postoperative Day 5.

## 3 DISCUSSION

An internal abdominal hernia refers to the protrusion of internal organs through a weak abdominal tissue wall. A transmesenteric hernia is an internal abdominal hernia in which there is an abnormal movement of abdominal organs, mainly small intestinal loops, through the natural orifice or defects of the peritoneum or mesentery. It is among the causes of intestinal obstruction, which is a type of closed-loop intestinal obstruction, which can lead to intestinal ischemia, necrosis, perforation, peritonitis, and death [2, 7].

**Fig. (3):**
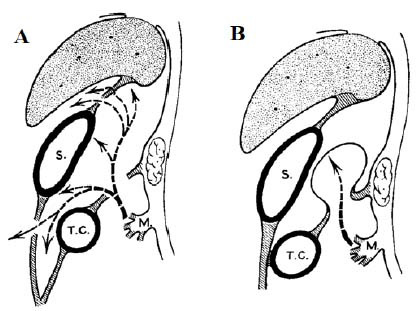
Illustration of the hernia mechanism through the transverse colon mesentery [5]. **A**: Hernia with an orifice in the transverse colon mesentery. **B**: Hernia without perforation of the mesenteric sac wall. M = Mesenteric root of the bowel loop, S = Stomach, T. C. = Transverse colon

Our two reported cases involved intestinal inflammation/obstruction caused by an internal hernia through the TCM’s natural orifice. This type of hernia accounts for approximately 8% of internal hernia cases. The disease is divided into two groups depending on the degree of natural defect in the TCM: (1) a perforation of both the anterior and posterior layers of the TCM through which the intestinal loop passes completely and is often seen in adults, such as the patient in our second case (Fig. **3A**), (2) and a perforation of the posterior layer of the TCM but with an intact anterior layer; in this case, only a small intestinal loop usually passes through the perforation and protrudes into the anterior layer of the TCM similar to our first case (Fig. **3B**) [3-5]. The pathogenesis of this type of hernia remains unclear, but most hypotheses suggest that the cause is often a mesentery defect in which the mesentery becomes thinner or a hole is formed. This is often caused by a lack of blood supply, and the colon becomes compressed during movement in the fetal period [8-10].

Defects in the TCM appearing in different locations can cause the hernia to vary in location; therefore, the clinical symptoms of hernia are often atypical or nonspecific, and sometimes, the predominant symptoms are those associated with an intestinal obstruction. Symptoms of hernia often progress over time as a result of a closed-loop intestinal obstruction. In many cases, due to the late diagnosis, there are manifestations of intestinal necrosis, perforation, and peritonitis [2, 3, 11]. In our first case, an internal hernia was diagnosed on the second day of illness, mainly based on the imaging finding of enteritis that was causing abdominal pain and fever. The second case was diagnosed on the 10th day of illness because the patient described symptoms identical to those of gastroesophageal reflux or gastric ulcers, as in the cases reported by Bandara *et al*. (2021) [12]. Some other authors also believe that the hernias that pass through the Winslow foramen next to the duodenum and through the mesentery near the Treitz corner also often cause symptoms similar to gastric reflux due to increased stomach pressure, leading to late diagnosis [12-14].

Due to the atypical clinical symptoms and the short time between intestinal obstruction and necrosis, imaging studies play a crucial role in the early diagnosis and treatment of patients. Consequently, MDCT has become the first-line imaging technique for evaluating abdominal emergency pathology in general and internal hernia pathology in particular. Many studies have shown that MDCT has a sensitivity of 94%-100% and a specificity of 90%-95% in diagnosing intestinal obstruction [13]. With a thin-slice thickness and high spatial resolution, MDCT helps to accurately assess the location and type of the internal hernia, cervical morphology, depth of the hernial sac, components, and condition of the internal structures, thereby providing timely and correct diagnosis for the patient. With multiplanar reconstruction, MDCT is very helpful in visually displaying the pathological condition, thereby enabling good planning for the surgical treatment of the patient. Without contrast, MDCT helps detect signs of increased density in the intestinal wall, indicating the presence of an intramural hematoma. With contrast, MDCT is useful for diagnosing the cause and severity of intestinal obstruction, specifically in the following ways: (1) In the arterial phase, it assesses blood flow, which helps determine the condition of a small intestinal obstruction; evaluates the path of blood vessels in relation to adjacent structures; and identifies blood vessels passing through the hernia orifice, thereby aiding in the diagnosis and classification of internal hernias. (2) In the portal venous phase, MDCT has an important role in evaluating the status of the small intestine, mesentery, presence of thrombosis, or gas in the mesenteric or portal veins [1, 6]. Small bowel obstruction caused by an internal hernia is usually a closed-loop bowel obstruction, that is, a bowel loop that is strangulated at both ends [6, 13, 15]. The direct sign of a closed-loop bowel obstruction on MDCT is a visualization of a U- or C-shaped loop of the bowel, with fluid-fluid level, distended bowel loops, mesenteric thickening centripetal, and a spoke- or fan-shape [1, 6].

Over time, MDCT has almost become the gold standard for diagnosing small bowel obstruction; however, with transverse mesocolic internal hernia, it is sometimes difficult to make a definitive diagnosis before surgery. In diagnosing transverse mesocolic internal hernia, MDCT has a sensitivity of 63%, specificity of 76%, and accuracy of 77% [11]. According to Blachar (2001), MDCT images suggestive of a transverse mesocolic internal hernia often show a U- or C-shaped closed loop of the intestine passing through the TCM upward and pushing the transverse colon forward, which displaces the mesenteric vascular bundle to the right of the inferior vena cava accompanied by high intestinal obstruction. Depending on when the illness occurs, there are different changes inside the hernial sac and surrounding tissue; if it occurs late, the closed intestinal loop is dilated, the intestinal wall is thickened, and there is fluid and infiltration in the hernial sac. There are three signs to be aware of: transverse colonic displacement, displacement of the mesenteric vascular bundle, and a closed loop of the intestine passing through the TCM, lying posteriorly that is found in >90% of cases. According to Blachar (2001), although all imaging criteria mentioned above were applied, correct diagnosis for this type of hernia before surgery only occurred in 2/14 (14.3%) patients [2, 6, 11, 16]. In the patient in our second case, although there were full imaging signs preoperatively, the final diagnosis was still a left para-duodenal internal hernia, which proves that the correct diagnosis of internal hernia before surgery is quite difficult.

Factors, such as the patient’s clinical presentation, progression of volvulus, and intestinal ischemia, depend largely on the size of the mesenteric defect (usually approx. 2-5 cm), herniated bowel-loop length, and the status of the surrounding structure [3]. The use of MDCT with multiplanar reconstruction helps to clearly reveal the morphology of the mesenteric defect [6].

After the diagnosis is confirmed, the treatment for a transverse mesocolic internal hernia is to perform emergency surgery as soon as possible to release the hernia and close the mesenteric defect to prevent recurrence. In the patient in our first case, the mesenteric defect was closed with continuous sutures, and in the patient in the second case, interrupted sutures were used. The choice of suture type was based entirely on the surgeon's preferences and experience. The surgeon explained that in the first case, continuous sutures were employed to close the anterior layer of the mesentery, as the posterior layer of the TCM remained intact, thereby avoiding the creation of a secondary diverticulum. In the second case, both the anterior and posterior layers of the TCM were perforated, and the herniated bowel loop was strangulated at the neck area. In the open surgery, our surgeons widened the mesenteric defect to free the intestinal loop, applied warm gauze to enhance the circulation to nourish it, continued to monitor for preservation, and then performed intermittent sutures to close the mesenteric defect while avoiding damage to the surrounding blood vessels. Furthermore, in the case of irreversible ischemia, resection of the intestinal segment and re-establishment of circulation are inevitable [2, 10, 17].

## CONCLUSION

The location of an internal hernia passing through the natural orifice in the TCM is inconsistent. Diagnosis based on clinical examination is quite difficult because this type of hernia can easily be confused with other types of internal hernias. However, imaging using MDCT with multiplanar reconstruction helps to diagnose the condition, determine the cause, and assess the state of intestinal ischemia. Moreover, early diagnosis and timely treatment are important for reducing complications and mortality rates in patients.

## Data Availability

All data generated or analyzed during this study are included in this published article.

## LIST OF ABBREVIATIONS

CLB: Closed loop of the bowel
D: Duodenum
M: Mesenteric
MDCT: Multidetector-row computed tomography
S: Stomach
SMA: Superior mesenteric artery
SMV: Superior mesenteric vein
TC: Transverse colon
TCM: Transverse colon mesentery

## STANDARDS OF REPORTING

CARE guidelines were followed.
